# Description and results of a new method for assessing real-life performance of a UV-C disinfection robot

**DOI:** 10.1016/j.infpip.2023.100322

**Published:** 2023-11-01

**Authors:** Michael Rodgers, Suzan Cremers, Edmée Bowles

**Affiliations:** Department of Medical Microbiology, Infection Prevention and Control Unit, Radboud Centre for Infectious Diseases, Nijmegen, Netherlands

**Keywords:** UV-C, Robot, Disinfection, Patient room

## Abstract

**Background:**

Due to the disadvantages of manual disinfection of patient rooms, mobile disinfection robots using ultraviolet C (UV–C) radiation are increasingly being used. Assessing their in situ effectiveness remains challenging.

**Aim:**

This study describes a new method to prove adequate in situ disinfection (≥5-log reduction in bacterial load), and uses this method to assess the efficacy of a mobile disinfection robot using UV-C radiation.

**Methods:**

Agar plates serving as proxies for smooth surfaces in patient rooms were inoculated with bacterial suspension and placed on various surfaces in a patient room. After irradiation by an automated mobile UV-C robot, reduction in colony growth was determined by comparing the irradiated plates to a reference series of non-irradiated plates, enabling the evaluation of whether an adequate reduction in colony-forming units (CFU's) of ≥5-log was reached on these irradiated surfaces.

**Findings:**

The new technique described here proved a successful method for demonstrating an in situ ≥5-log reduction in CFU's for five different bacterial pathogens. Of the 32 plates placed on UV-accessible surfaces, 31 showed an adequate reduction in CFU's of ≥5-log. One plate could not be assessed.

**Conclusion:**

Inoculated agar plates placed in patient rooms before irradiation and subsequently compared to a reference series can be used to assess in situ efficacy of mobile disinfection robots using UV-C radiation. Our findings support the idea that UV-C robots, used adjunctively to conventional manual washing and disinfection, may achieve adequate bacterial load reduction on UV-accessible smooth surfaces in patient rooms for a selected subset of pathogens.

## Introduction

Cleaning and disinfection of hospital patient rooms after use by patients carrying multidrug resistant micro-organisms (MDRO) is an important aspect of infection prevention and control. However, the process of disinfection is labour-intensive, difficult to standardize and therefore prone to human error, and may require the use of disinfectants detrimental to the environment.

A number of alternatives for, or adjuncts to, disinfection are available, one of which is disinfection utilizing ultraviolet C (UV–C) radiation. UV-C might be used to replace manual disinfection of surfaces in patient rooms accessible to UV-C irradiation, after manual cleaning of these surfaces has been performed. UV-C kills organisms by preventing DNA-replication [1]. Questions regarding its effectiveness in real-life settings remain. Multiple studies, both in vitro, and in real-life (in situ) settings, have been performed to assess the effectiveness of UV-C radiation in reducing bacterial load on surfaces. A recent systematic review collated these in situ studies [2]. Although all of these studies showed in situ effectiveness of UV-C in reducing bacterial load on patient room surfaces, none described a technique with which a 100,000-fold (5-log) reduction in bacterial load could be demonstrated*.* Demonstrating a 5-log reduction is important, since adequate disinfection is usually defined by a 5-log reduction in bacterial load. [3].

Therefore, in this study we present an alternative method to assess in situ bacterial load reduction using UV-C. This method does allow quantification of (at least) a 5-log reduction, using Rodac plates as surrogates for patient room surfaces. We also present the results of using this method to assess the effectiveness of reducing bacterial load on UV-accessible surfaces in a hospital patient room using an automated UV-C robot.

## Methods

### Irradiation procedure

For this study we used the SAM UV-C robot, which is an automated, mobile UV-C robot manufactured by Loop Robots B.V. in the Netherlands. [4] It utilizes several 253.7 nm germicidal light sources situated at various locations on the robot. The robot was operated in fully autonomous mode to disinfect the patient room and the connected anteroom with airlock with the door between room and anteroom left open (at 45 degrees). The aim of the robot as stated by the manufacturer is to provide adequate disinfection of unshaded smooth surfaces in the patient room below elbow height (from now on referred to as UV-accessible surfaces). It is programmed to deliver at least a dosage of 30 mJ/cm^2^ on these UV-accessible surfaces.

The execution of this automated program took 16 minutes. The sanitary space, (toilet, wash basin and shower), which is connected to the room, was disinfected in a separate program cycle of 8 minutes.

### Assessing effectiveness of disinfection

Adequacy of disinfection was assessed using Rodac plates (containing tryptone soy agar, tween 80 and lecithine, provided by Balis Laboratorium B.V.) directly inoculated with bacteria. Rodac plates were specifically chosen because the rim of these plates is flush with the agar, in contrast to most conventional plates which have elevated rims which can block UV-light. The strains used were *Escherichia coli* ATCC 25922, *Staphylococcus aureus* ATCC 29213, *Enterococcus faecium* (a clinical isolate producing the resistance enzyme VanB), *Pseudomonas aeruginosa* (ATCC 27853) and *Acinetobacter baumannii* (clinical isolate). These strains were chosen as they are likely to be similar in UV susceptibility to equivalent multi-drug resistant strains.

A total of 50 test plates were used, 11 containing *E. coli*, 10 containing *S. aureus*, 10 containing *E. faecium*, 9 containing *P. aeruginosa*, and 10 containing *A. baumannii*.

**Fig S6:**
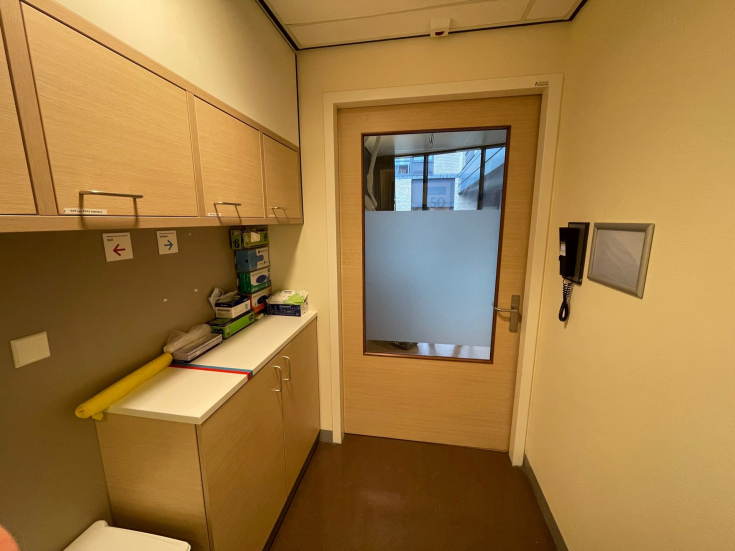


These plates were all placed in the patient room at different positions. See supplementary figures A1-6 for an overview of the patient room. The goal was to cover a wide and diverse array of surfaces in the room with test plates. Examples of the surfaces tested were the counter, the inside of the washbasin, keyboard, chair, outside surface of drawer of nightstand, divider wall, surface on door behind door handle, backrest of chair, top of tissue dispenser and shower rail (see Table I). Thirty-two plates were placed on UV-accessible surfaces (i.e. unshaded and below elbow height). Eighteen plates were placed on surfaces which were (partly) shaded and/or above elbow height (surfaces with limited UV-accessibility).

**Table I:**
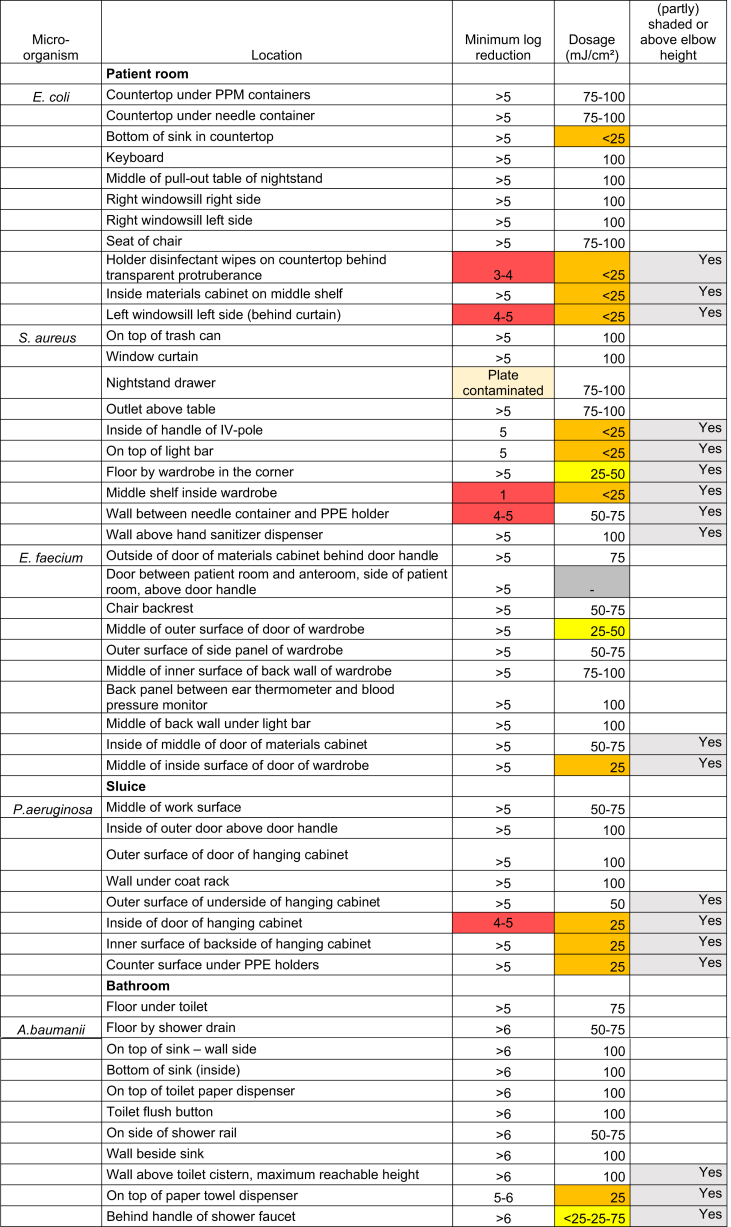
Individual results. Overview showing the tested plates, the micro-organisms used, the surfaces they were placed on, the demonstrated log-reduction, the dosage received per surface, and whether the plates were placed on UV-accessible surfaces (unshaded and below elbow height), or not

**Figure 1:**
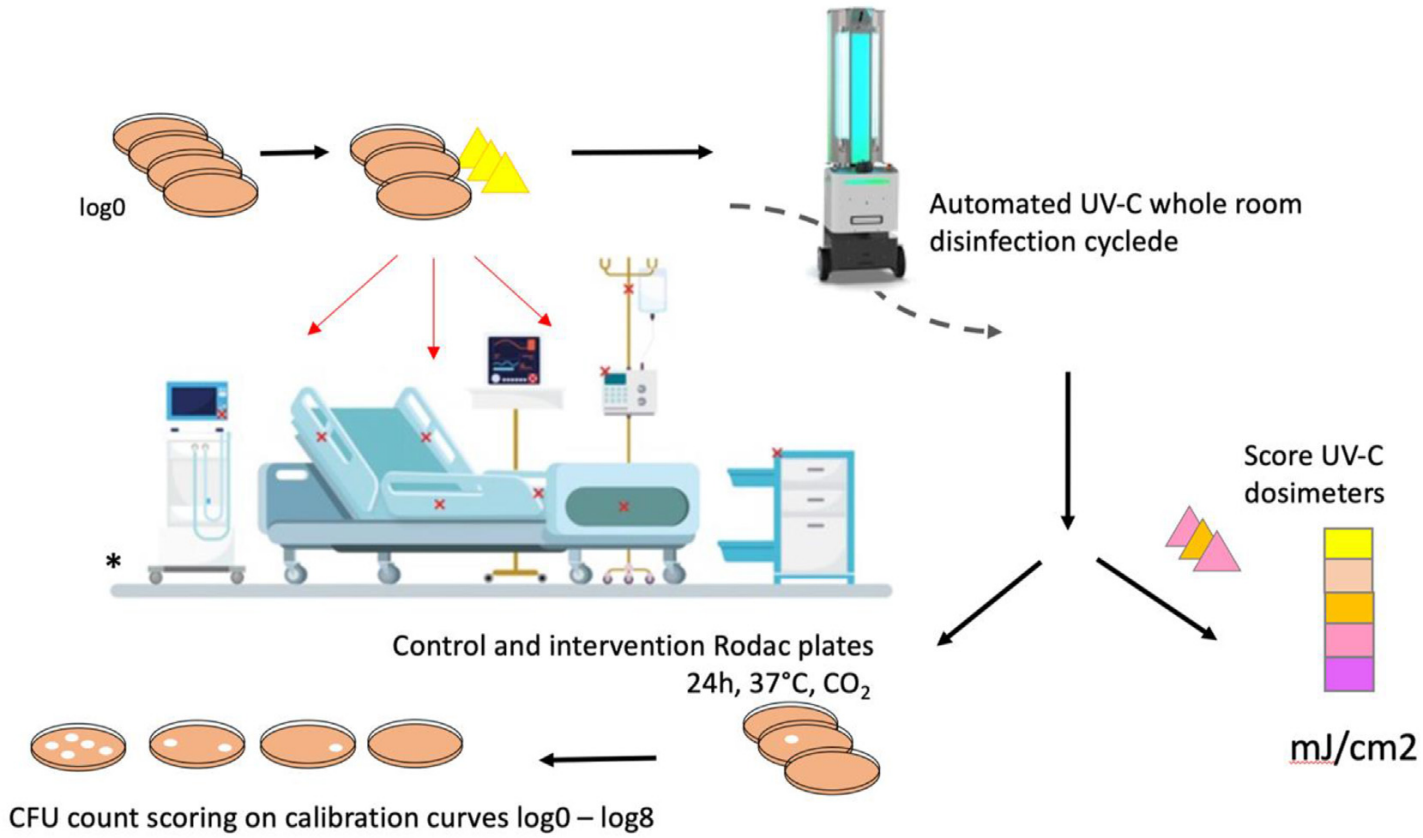
Summary of the process. Schematic representation of the placement of the test plates and dosimeters in the patient room, and subsequent comparison of the test plates to the reference series.

The UV-C dosage (measured in millijoules per square centimeter [mJ/cm^2^]) per surface was estimated through the use of dosimeter indicator strips (Intellego Technologies). These strips were placed next to the sample, and change colour depending on the total exposure to UV-C. They are colour coded to show exposure to dosages of <25, 50, 75 and 100 mJ/cm^2^. See Figure 1.

After the disinfection cycle, the reduction in bacterial load on each plate was determined using the method described below. A reduction of at least 5-log was classified as adequate reduction. A reduction of less than 5-log was classified as inadequate reduction.

The primary endpoint was the proportion of test plates placed on UV-accessible surfaces which showed adequate reduction in bacterial load. As a secondary endpoint we looked at test plates placed on surfaces with limited UV-accessibility. Additionally, we used dosimeters to determine the proportion of surfaces which received an adequate dosage of UV-C according to the dosimeters.

### Preparation and assessments of Rodac plates

Figure 1 shows a visual summary of the process. In short, to assess the reduction in bacterial load after room disinfection with the UV-C robot, we inoculated Rodac plates with a suspension of the pathogen. These plates (the ‘test plates’) were placed on different surfaces and at different angles in the room which was to be disinfected. After the disinfection cycle was performed by the UV-C robot, the plates were incubated for 24 hours at 36 degrees at 5% CO2, together with a reference series consisting of a serial dilution of the pathogen. After incubation, the bacterial density on the test plates was compared to the reference series to determine the reduction of bacterial load on the test plates.

### Creating a reference series

For each organism, a 0,5 McFarland suspension was made in 0.9% saline. This suspension was used to inoculate both the Rodac plates which were to be irradiated, as well as the first plate in the reference series. Inoculation of the plate was performed using a cotton swab, covering the test plate three times at 60 degree angles, according to the Kirby Bauer method. Subsequently, the suspension was diluted tenfold (creating a 1-log dilution), after which the second plate in the reference series was inoculated. This dilution was again diluted tenfold (creating a 2-log dilution), and inoculated onto the third plate in the reference series. These steps were repeated until an 8-log dilution was reached.

**Figure 2:**
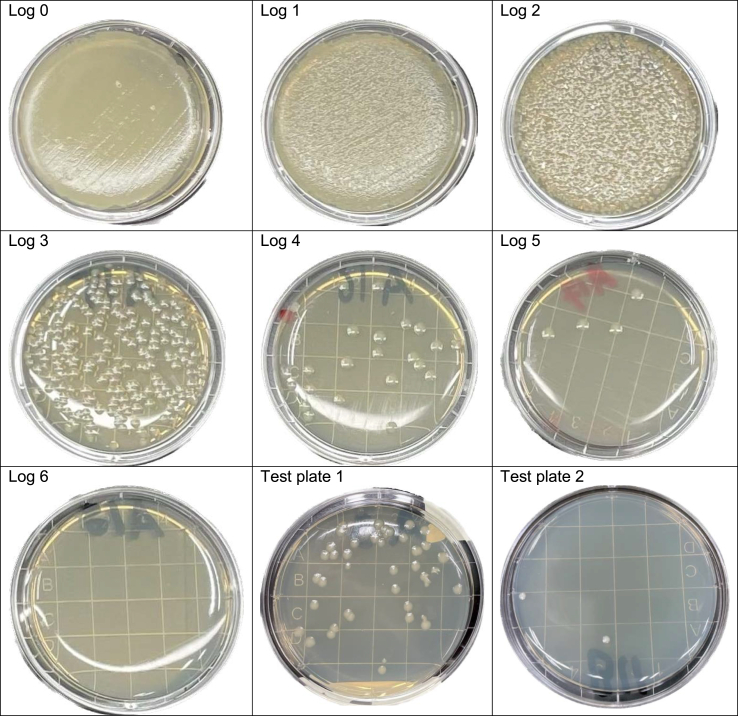
Reference series for E. coli and two test plates. Example of a reference series with growth up to 5-log dilution, meaning that a maximum reduction of ≥log 5 can be proven. Example: test plate 1 shows a reduction of 3-4-log. Test plate 2 shows a reduction of >log 5.

This way, a reference series was created with each plate harbouring a tenfold lower load of organisms compared to the previous plate (see Figure 2). The reference series was kept outside of the patient room, and incubated for 24 hours, together with the test plates (after they had been placed in the patient room and the disinfection cycle had been completed). After 24 hours, the growth on the test plates was compared to the growth on the plates of the reference series, and the number of CFU was determined. A ≥5-log reduction could be proven if the test plate showed growth of an equal number of, or fewer colonies than, the reference plate containing the 5-log dilution.

## Results

### Reference series

The reference series for *E. coli*, *S. aureus* and *P. aeruginosa* showed growth up to and including the 5-log dilution plate, meaning that this setup allowed demonstration of a ≥5-log reduction for these pathogens. The reference series for *E. faecium* and *A. baumannii* showed growth up to and including the 6-log dilution plate, meaning that for these organisms a maximum of ≥6-log reduction could be proven with this setup.

### Demonstrated reduction in CFU's

Of the 32 plates which were placed on UV-accessible surfaces, 31 reached the primary endpoint of showing an adequate reduction in CFU's of ≥5-log. One plate could not be interpreted due to contaminating growth. Of these 32 plates, 29 (91%) were shown to have received an adequate UV-C dosage of ≥50 mJ/cm^2^. For one plate the dosage was not measured. See Table II.

**Table II:**
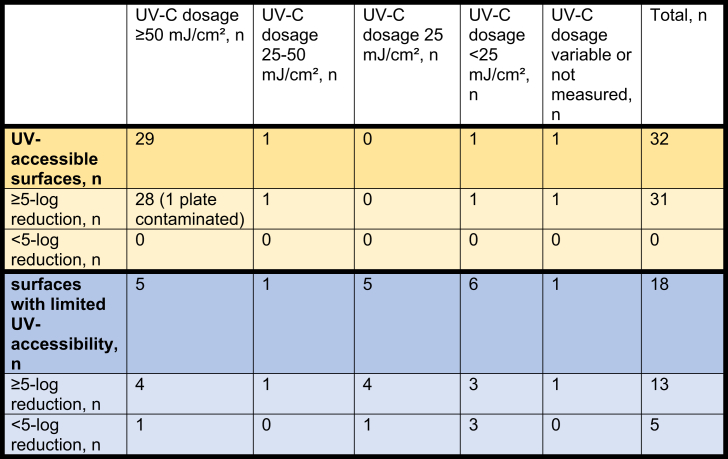
Overview of UV-C dosage received per plate, and reduction of CFU's per plate. The results are presented separately for the plates showing ≥5-log and <5-log reduction respectively. Results are split between plates placed on UV-accessible surfaces (unshaded surfaces below elbow height), and plates placed on surfaces with limited UV-accessibility ([partly] shaded and/or above elbow height)

Of the 18 plates which were placed on surfaces with limited UV-accessibility, 13 showed an adequate reduction in CFU's of ≥5 log. Of the five plates which showed inadequate reduction in CFU's, two contained E. coli, two contained *S. aureus*, and one contained *P. aeruginosa*, see Table I. Of these 18 plates, five were shown to have received an adequate UV-C dosage of ≥50 mJ/cm^2^. The dosimeter for one plate showed a variable result, with the dosimeter showing less colour change closer to the source of the shadow.

## Discussion

This study presented an as of yet undescribed method for determining in situ adequacy of disinfection using UV-C. Previous studies have looked at in situ effectiveness in reducing bacterial load, but none described a method which could be used to prove a ≥5-log reduction. Three studies directly inoculated surfaces and cultured these surfaces after irradiation [[5], [6], [7]]. Three studies cultured directly from patient room surfaces by inoculating Rodac plates through direct contact with these surfaces [[8], [9], [10]]. Two studies took direct cultures from patient room surfaces using swabs [7,11]. One study did not describe how they were able to quantify the demonstrated reduction [12]. As these previous studies show, demonstrating a ≥5-log reduction by culturing directly from patient room surfaces is hardly possible. The bacterial load and subsequent recovery rate of bacteria from these surfaces is too low. The number of colony forming units (CFU's) recovered from the control (i.e. non-irradiated) surfaces never reaches 100,000, while recovery of at least 100,000 CFU's from the control surface would be needed to be able to demonstrate a 100,000-fold reduction on the irradiated surfaces.

The protocol described here uses directly inoculated Rodac plates, which are placed in the test environment and are directly exposed to the UV-C. The advantage hereof is that a far greater reduction in bacterial load can be proven as compared to previously described methods. This is due to the fact that the step of inoculating an object, and subsequently culturing of that object, is avoided. Inoculating off an object (either by directly applying a Rodac plate to that object, or by washing the object and culturing the fluid) leads to a loss of CFU's, reducing the sensitivity of the procedure, making it nearly impossible to prove a 5-log reduction in bacterial load.

A limitation of the method described here is the fact that the micro-organisms used here were all fast-growing organisms, abundantly growing on Rodac agar. To use this method with slower growing or more fastidious organisms, such as Candida species, a longer incubation time and a higher initial inoculum might be needed to be able to create a reference series which still shows growth on the 5-log dilution plate. For this to be achieved, a higher volume of fluid and a higher concentration of micro-organisms might be used when inoculating the plates.

Regarding the effectiveness of the UV-C robot tested in this study, adequate disinfection was found on all UV-accessible surfaces tested, thereby offering support for the idea that this system might be able to provide adequate disinfection of smooth UV-accessible surfaces below elbow height in patient rooms. These surfaces would however still require manual washing before disinfection, and other surfaces would still require conventional disinfection approaches. An adequate dosage of UV-C was demonstrated on nearly all of the UV-accessible surfaces. The two UV-accessible surfaces which were not shown to have received an adequate dosage of UV-C nevertheless showed an adequate reduction in CFU of ≥5-log. Adequate disinfection was also seen on some of the surfaces with limited UV-accessibility.

Inadequate disinfection was, as expected, found on surfaces including insides of open cupboards, behind handles and behind a transparent plastic protuberance. It therefore remains important to identify potential shaded areas and areas above elbow height should this UV-C robot be used, and to perform manual disinfection of these areas.

A limitation of this study is that Rodac plates were used as surrogates for patient room surfaces. Rodac plates are smooth-surfaced, as are most surfaces in a patient room. However, surfaces which are not smooth, such as fabric, or materials which contain creases, such as curtains, create (micro)-shadows, possibly leading to inadequate irradiation and disinfection of these materials. Additionally, a limitation is that one micro-organism was tested per surface, instead of testing all five micro-organisms on all surfaces, which was not feasible in this study. Moreover, only a small selection of the broad range of potential pathogens were tested, all of them bacteria. Potential other pathogens which warrant specific testing include yeasts such as Candida auris, mycobacteria such as Mycobacterium tuberculosis, and spore-forming bacteria such as *Clostridium difficile*. Previous studies did show reduction of *C. difficile* [[5], [6], [7], [8], [9], [10], [11]], *Mycobacterium abscessus* [12], and *Candida* species [7] using UV-C. However, these studies were not designed to demonstrate a ≥5-log reduction. Therefore further research will need to determine if real-life use of UV-C also leads to adequate reduction in the load of these organisms.

Viruses form a group of pathogens for which our method cannot be used to demonstrate adequate disinfection. We did not find any studies examining the in situ efficacy of UV-C in reducing viral contamination of surfaces in patient rooms. Several in vitro studies did show effectiveness of UV-C in reducing viral load on surfaces [13], with one in vitro study showing a >log 5 reduction in active virus [14]. They however did not specify the wavelength and dosage of UV-C used. A recent study did examine the in vitro effectiveness of different combinations of wavelengths and dosages of UV-irradiation on inactivation of a number of viruses, and concluded that with short wavelengths (255 and 265 nm, similar to the 253.7 nm used here), 90% inactivation was achieved at dosages <15 mJ/cm^2^ [15]. In our study, significantly higher dosages were achieved on nearly all of the UV-accessible surfaces. Another study simulated an in situ situation by using a simulated hospital room and used a mobile autonomous UV-C robot emitting 254 nm rays [16]. They achieved a reduction in viable SARS-CoV-2 virus of ≥99.91% to ≥99.99%. However, the dosage per cm^2^ may have been higher (50–500 mJ/cm^2^) than the dosages achieved in this study, as the dosimeters used in our study were not able to quantify dosages above 100 mJ/cm^2^. To summarize, there is evidence also suggesting effectiveness of UV-C against viral contamination of surfaces, but in situ studies are needed to further prove this.

## Conclusions

This paper demonstrates a new method to quantify reduction in bacterial load (for fast-growing bacteria) of at least 5-log for different locations in hospital patient rooms using UV-C, by using direct placement of inoculated Rodac plates in the patient room during disinfection and subsequent comparison to a reference series of plates.

The results of this in situ study using the above method support the idea that UV-C robots can be used to achieve an adequate reduction in bacterial load of ≥5-log on smooth UV-accessible surfaces within patient rooms and may therefore have a role as an adjunctive method of patient room disinfection. However, more studies are needed to confirm the safety and applicability of this system. In this study only a small subset of potential pathogens was tested. This data cannot necessarily be extrapolated to other potential hospital pathogens such as *M. tuberculosis*, *C. difficile*, *Candida* species and viruses. For these pathogens, additional in situ studies are needed. Areas which are shaded to the UV-C rays produced by the robot, including areas behind transparent objects, need to be identified when implementing this device, and will still require manual disinfection. Lastly, manual cleaning will always be needed prior to disinfection, to remove visible surface contamination and thereby enable adequate disinfection.

## Acknowledgements

None.

## Credit author statement

Conceptualization was done by MR, SC and EB. Methodology: MR developed the new method for determining in situ efficacy. SC and EB determined how to test this in practice with a mobile UV-C robot. Analysis of the results was performed by SC and MR. Writing of the original draft was performed by MR. Reviewing and editing of the draft was done by SC and EB. Visualization was done by MR. Supervision was done by EB.

## Conflict of interest statement

None.

## Funding statement

No funding was received for this study.

## Ethics statement

Not required as this study did not involve patients or patient materials.

## Figures and Tables

**Fig S1:**
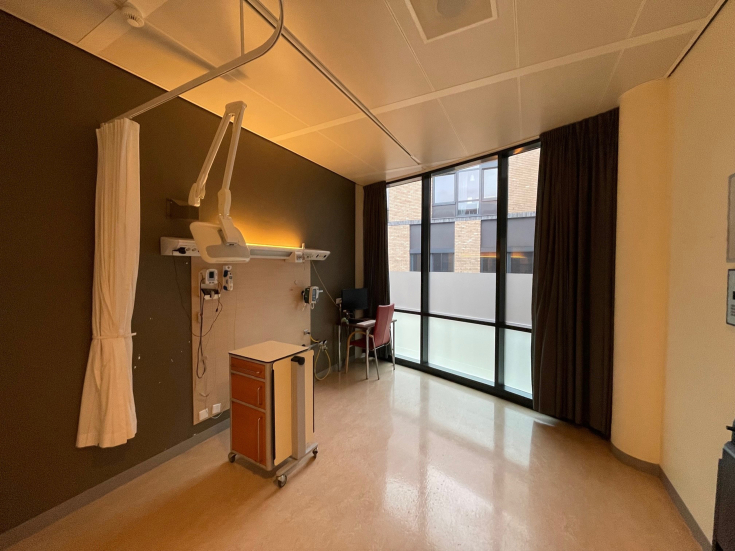


**Fig S2:**
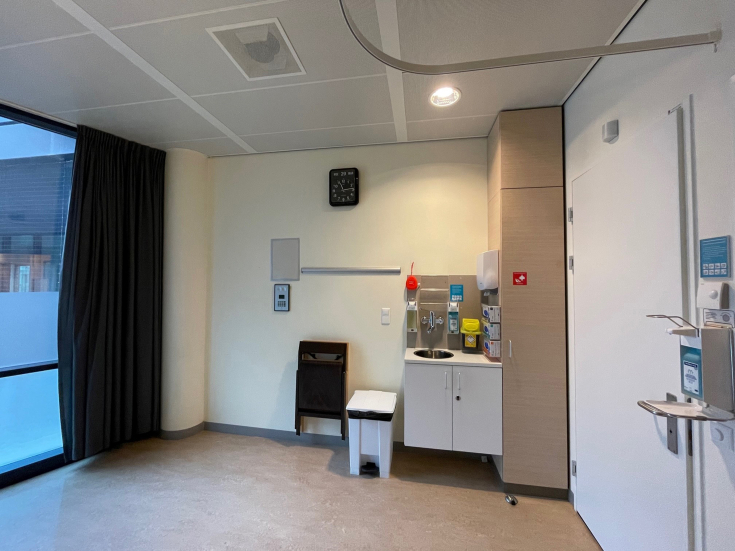


**Fig S3:**
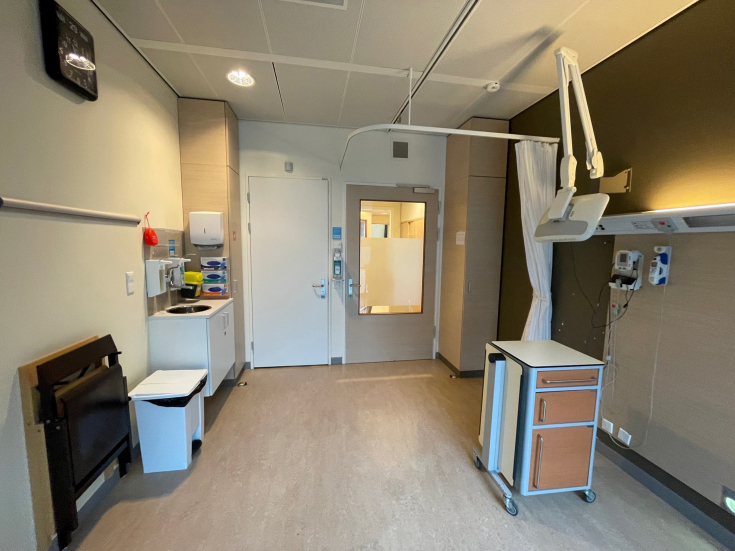


**Fig S4:**
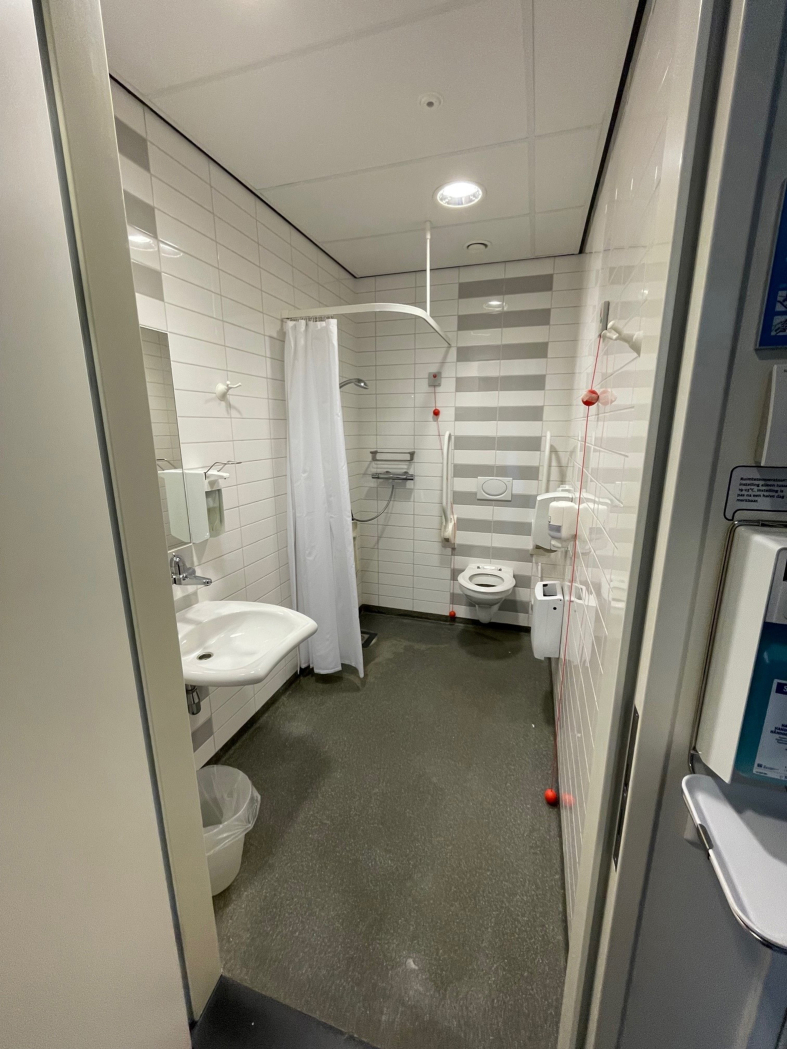


**Fig S5:**
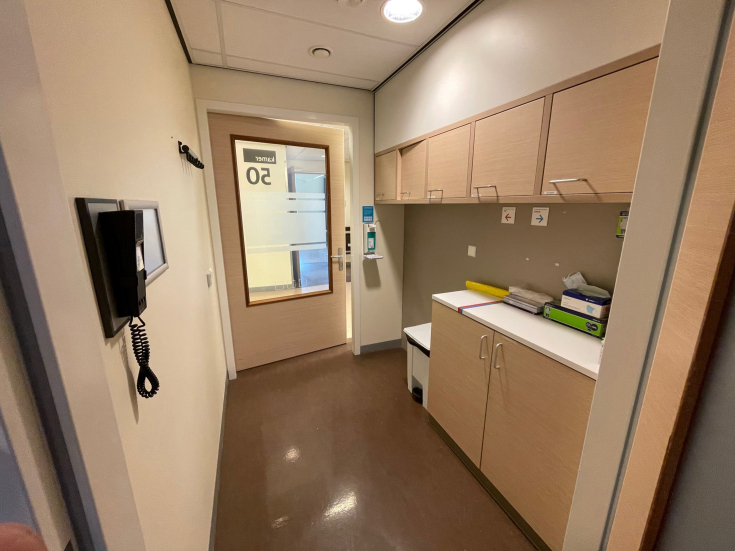


## References

[1] Steele M., Hurtado R.R., Rychlik K., Bonebrake A., Bovee M.C., O'Donnell A. (2021). Impact of an automated multiple emitter whole-room ultraviolet-C disinfection system on hospital acquired infections: A quasi-experimental study. Am J Infect Control.

[2] van der Starre C.M., Cremers-Pijpers S.A.J., van Rossum C., Bowles E.C., Tostmann A. (2022). The in situ efficacy of whole room disinfection devices: a literature review with practical recommendations for implementation. Antimicrob Resist Infect Control.

[3] Gebel J., Exner M., French G., Chartier Y., Christiansen B., Gemein S. (2013). The role of surface disinfection in infection prevention. GMS Hyg Infect Control.

[4] https://www.looprobots.com/.

[5] Nerandzic M.M., Cadnum J.L., Pultz M.J., Donskey C.J. (2010). Evaluation of an automated ultraviolet radiation device for decontamination of Clostridium difficile and other healthcare-associated pathogens in hospital rooms. BMC Infect Dis.

[6] Rutala W.A., Gergen M.F., Weber D.J. (2010). Room Decontamination with UV Radiation. Infect Control Hosp Epidemiol.

[7] Mustapha A., Alhmidi H., Cadnum J.L., Jencson A.L., Donskey C.J. (2018). Efficacy of manual cleaning and an ultraviolet C room decontamination device in reducing health care–associated pathogens on hospital floors. Am J Infect Control.

[8] Anderson D.J., Gergen M.F., Smathers E., Sexton D.J., Chen L.F., Weber D.J. (2013). Decontamination of Targeted Pathogens from Patient Rooms Using an Automated Ultraviolet-C-Emitting Device. Infect Control Hosp Epidemiol.

[9] Rutala W.A., Kanamori H., Gergen M.F., Knelson L.P., Sickbert-Bennett E.E., Chen L.F. (2018). Enhanced disinfection leads to reduction of microbial contamination and a decrease in patient colonization and infection. Infect Control Hosp Epidemiol.

[10] Wong T., Woznow T., Petrie M., Murzello E., Muniak A., Kadora A. (2016). Postdischarge decontamination of MRSA, VRE, and Clostridium difficile isolation rooms using 2 commercially available automated ultraviolet-C–emitting devices. Am J Infect Control.

[11] Liscynesky C., Hines L.P., Smyer J., Hanrahan M., Orellana R.C., Mangino J.E. (2017). The Effect of Ultraviolet Light on Clostridium difficile Spore Recovery Versus Bleach Alone. Infect Control Hosp Epidemiol.

[12] Yang J.-H., Wu U.-I., Tai H.-M., Sheng W.-H. (2019). Effectiveness of an ultraviolet-C disinfection system for reduction of healthcare-associated pathogens. J Microbiol Immunol Infect.

[13] Viana Martins C.P., Xavier C.S.F., Cobrado L. (2022). Disinfection methods against SARS-CoV-2: a systematic review. J Hosp Infect.

[14] Bedell K., Buchaklian A.H., Perlman S. (2016). Efficacy of an Automated Multiple Emitter Whole-Room Ultraviolet-C Disinfection System Against Coronaviruses MHV and MERS-CoV. Infect Control Hosp Epidemiol.

[15] Schöbel H., Diem G., Kiechl J., Chistè D., Bertacchi G., Mayr A. (2023). Antimicrobial efficacy and inactivation kinetics of a novel LED based UV-irradiation technology. J Hosp Infect.

[16] Lorca-Oró C., Vila J., Pleguezuelos P., Vergara-Alert J., Rodon J., Majó N. (2022). Rapid SARS-CoV-2 Inactivation in a Simulated Hospital Room Using a Mobile and Autonomous Robot Emitting Ultraviolet-C Light. J Infect Dis.

